# Supplementary material for: Cladoceran Chydorus sphaericus and Colonial Cyanobacteria: Potentially a Toxic Relationship?
Source: Toxins (Basel). 2025 Jun 12;17(6):298. doi: 10.3390/toxins17060298 (PMC12197402; doi:10.3390/toxins17060298)
Supplement: Supplementary file 1 [file toxins-17-00298-s001.zip › toxins-3616229-supplementary.pdf]

Supplementary Material

Cladoceran *Chydorus sphaericus* and colonial cyanobacteria: Potentially a toxic relationship?

Helen Agasild, Ilmar Tõnno, Margarita E. Gonzales Ferraz, Peeter Nõges, Priit Zingel, Lea Tuvikene, René Freiberg, Tiina Nõges, Kristel Panksep

Supplementary Table S1. Water quality parameters in Lake Võrtsjärv sampling sites in 2015.

**Sampling Site**	**10**		**1**	**2**	**3**	**4**	**5**	**6**	**7**
**Variables**	**Mean**	**Range**	**Mean**	**Mean**	**Mean**	**Mean**	**Mean**	**Mean**	**Mean**
TP, µg/L	41	29-58	46	37	37	40	70	55	75
TN, µg/L	1552	720-3000	950	930	1200	970	1300	1200	1200
NO_3^-, µg/L	710	20-2600	55	13	14	18	16	30	19
NO_2^-, µg/L	7	3-18	<3	<3	<3	<3	<3	<3	<3
NH_4^+, µg/L	45	20-110	<20	<20	<20	<20	<20	<20	<20
Water Temp, °C	8.6	0.2-19.4	19.3	19.3	19.3	19.6	19.6	19.6	20.0
Secchi depth, m	0.9	0.45-1.7	0.55	0.6	0.5	0.6	0.4	0.45	0.45
pH	8.35	7.9-8.7	8.51	8.65	8.66	8.66	8.67	8.79	8.84
O_2, mg/L	12.1	9.5-14.4	13.23	13.1	13.01	13.32	13.2	13.79	15.16
Chl-*a,* µg/L	34.6	1.2-61.9	44.3	34.3	30.9	37.1	72.9	52.8	62.3
